# The Effects of Fatigue on the Lower Limb Biomechanics of Amateur Athletes during a Y-Balance Test

**DOI:** 10.3390/healthcare11182565

**Published:** 2023-09-16

**Authors:** Congyu Huang, Jingyi Ye, Yang Song, Balint Kovács, Julien S. Baker, Zhuqing Mao, Yaodong Gu

**Affiliations:** 1 Faculty of Sport Science, Ningbo University, Ningbo 315832, China; 2 Doctoral School of Safety and Security Sciences, Obuda University, 1034 Budapest, Hungary; 3 Faculty of Engineering, University of Szeged, 6724 Szeged, Hungary; 4 Department of Kinesiology, University of Physical Education, Alkotás u. 44, 1123 Budapest, Hungary; 5 Centre for Health and Exercise Science Research, Hong Kong Baptist University, Hong Kong 999077, China; jsbaker@hkbu.edu.hk; 6 Department of Radiology, Ningbo No. 2 Hospital, Ningbo 315010, China

**Keywords:** Y Balance Test, fatigue, lower extremity, kinematic, dynamics

## Abstract

The Y-Balance Test (YBT) is a reliable tool for assessing the dynamic balance of athletes’ lower limbs. This study aimed to compare the effects of the YBT on lower limb biomechanics before and after fatigue. Sixteen adult male recreational athletes were recruited for the study, and motion capture in combination with a force plate was used to collect kinematic, dynamics, and center of pressure (COP) data of the dominant leg during YBT testing before and after fatigue. Based on the research findings, there were significant statistical differences in the distances reached during the YBT in three directions before and after fatigue. After fatigue, there is a significant decrease in the ROM of the hip and knee joints in all three directions. Also, there is a significant increase in hip joint torque in the anterior- and posterior-lateral directions, while a significant decrease in hip and ankle joint torque is observed in the posterior-medial direction. Moreover, there is an increasing trend in positive and negative joint work for the hip, knee, and ankle joints in all three directions after fatigue. The range of COP displacement also increases following fatigue. The decline in YBT scores demonstrates the detrimental impact of fatigue on the dynamic balance of the lower limbs of adult male amateur athletes. We hope that these results can provide information for athletes and coaches to better understand the effects of fatigue on the dynamic balance of lower limbs, so as to carry out targeted lower limb balance training and prevent sports injuries.

## 1. Introduction

Balance is a critical component of athletic performance and injury prevention for athletes, and a lack of balance is closely associated with an increased risk of lower limb injuries [1]. Therefore, balance is of utmost importance for athletes, as it significantly impacts the quality of their training and competitive performances [2]. Balance, as an ability, has been defined as comprising two conditions, static and dynamic balance. Static balance refers to the ability of the human body to maintain posture or stability and to control its center of gravity in a relatively stationary state [3,4,5]. Dynamic balance refers to the ability of the human body to automatically adjust and maintain posture and control balance when moving or being subjected to external forces [6]. Since most sports are performed under dynamic conditions, dynamic balance is one of the most important factors influencing daily activities and sports performance.

There are several field tests that exist to obtain data of the dynamic balance of an athlete, and the Y-Balance test (YBT) is one of the most widely accepted methods for assessing dynamic postural stability [7,8,9]. It is a cost-effective and commonly used objective measurement method for assessing lower extremity dynamic balance, functional symmetry, and stability [9,10,11,12]. The YBT can evaluate an individual’s performance in terms of muscle coordination, stability, and symmetry in three directions: anterior (A), posterolateral (PL), and posteromedial (PM). In the YBT, participants are required to maintain single-leg balance while reaching as far as possible with the other leg in three different directions. By observing the maximum reach distances in three directions and the degree of COP displacement, we can make a reasonable assessment of athletes’ dynamic balance capabilities [13].

Achieving balance requires the coordinated integration of sensory inputs, central processing, and motor control. Visual acuity, vestibular organs, the nervous system, muscle strength, and proprioception all play crucial roles in maintaining body balance [14,15,16]. Among these factors, the activity of the lower limb muscles is particularly critical for sustaining dynamic balance in athletes. Previous studies have shown that muscle fatigue is an important influencing factor in sports injuries and is also involved in decreases in dynamic balance ability [16,17,18]. Muscle fatigue refers to a decline in the ability of muscles to maintain or produce the expected force. Traditionally, muscle fatigue has been defined as a reduction in maximal strength or power induced by exercise [19].

Rose et al. [20] found significant differences in balance levels between fatigued and non-fatigued states among athletes. Both local and whole-body fatigue can result in decreased postural control performance, manifested as increased postural sway, which in turn disrupts the body’s balance and stability [21]. Previous studies have indicated that maximal anaerobic fatigue has a negative impact on athletes’ YBT scores [22]. Nader Abdelkader et al. [23] found that inducing fatigue during YBT measurements has inconsistent effects on the dynamic balance among different athletic populations. Following fatigue, the dynamic balance of professional athletes is better than that of amateur athletes. In the professional athletic population, a study by Ross Armstrong et al. [24] found that professional dancers’ YBT performances were not affected by fatigue induced by aerobic fitness testing (DATF). However, in the recreational sports population, Majid et al. [25] discovered that fatigue decreased all functional test scores and lower limb muscle activity levels in amateur athletes during the YBT. The findings demonstrated a significant impact of fatigue on dynamic balance in amateur athletes.

Currently, several studies have investigated the impact of lower limb fatigue on athletes’ balance abilities. However, there was a relative lack of specific research focusing on the changes in lower limb biomechanical parameters during the YBT in athletes. Therefore, the main objective of this study was to explore the biomechanical variations in the dominant supporting leg of adult recreational athletes during the YBT process, before and after inducing muscular fatigue. We hypothesized that fatigue would significantly influence the biomechanical characteristics of the lower limbs, particularly leading to increased hip and knee joint torques and increased work for the supporting leg. Additionally, we posited that fatigue might have also increased the length of the COP path of the lower extremities.

## 2. Materials and Methods

### 2.1. Participants

Effect sizes were calculated from previous research with methods that closely resembled this study [26]. Based on the a priori sample size calculation conducted using G*Power 3.1.7 [27], a minimum sample size of 16 participants was required to detect significant differences in YBT before and after fatigue (power: 0.8, effect size: 0.80, α = 0.05, and β = 0.2).

In this study, we recruited 16 recreational athletes (22 ± 1.8 yrs, 179.8 ± 3.4 cm, and 78.6 ± 6.9 kg) from the School of Physical Education at Ningbo University. These participants engaged in at least 1–2 h of physical exercise daily and had a history of regular sports training for at least 6 years, with 2–3 exercise sessions per week. To ensure the accuracy of the research results, we excluded individuals with a history of neurological or muscular diseases, lower limb fractures or surgeries within the past six months, and ankle sprains within the last year. All tests were conducted in the laboratory of the Faculty of Sports Science at Ningbo University. Prior to the study, the participants were provided with detailed information about the entire experimental procedure and all signed informed consent forms. The Ethics Committee of the Ningbo University Research Institute approved the study (RAGH202305013005.2), which was performed in accordance with the Declaration of Helsinki [28].

### 2.2. Experimental Design

**Figure 1:**
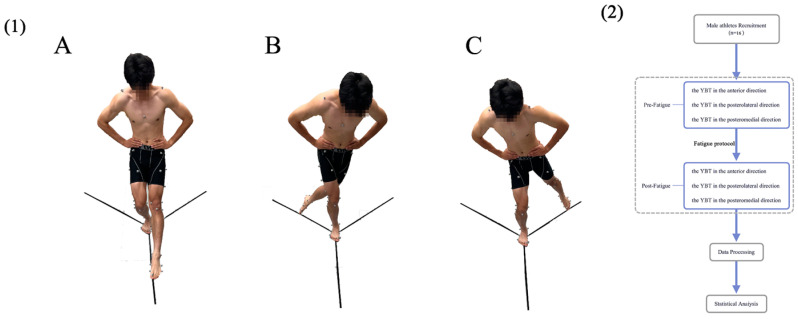
(**1**) The participants performing the Y-Balance test. (**A**) Participant demonstrating the Y-Balance Test in the anterior direction. (**B**) Participant demonstrating the Y-Balance Test in the posterolateral direction. (**C**) Participant demonstrating the Y-Balance Test in the posteromedial direction. (**2**) A flow chart was created to outline the data collection and processing.

Before the formal experiment, participants underwent a 5 min warm-up activity on a motorized treadmill. Participants were tested before and after performing the fatigue protocol. Before the measurement, the participants were familiarized with YBT by practicing the test on their dominant limb (determined by asking the participants which leg they would use to kick a ball). The YBT was conducted on a force plate, and black tape was used to mark the Y-shaped test positions on the force plate. The standing leg remained on the force plate during the whole task while the other leg performed error-free maximum arrival movements along the calibrated black tape in all three directions. The maximum distances reached in the anterior, posteromedial, and posterolateral directions related to the stance leg were recorded using a tape measure [29]. During the test, the participants kept their hands on their waists, and each direction was repeated three times with a 10 s rest in between each trial (Figure 1). After completing the fatigue protocol, the same testing procedures were repeated [25,30].

If the following situations occurred during the testing process, the participants needed to complete the test again: (1) the supporting leg deviates from the central area of the YBT system; (2) the heel of the supporting leg is off the ground; (3) instability of the center of gravity, leading to the reaching leg touching the ground; (4) the reaching leg cannot return smoothly to the starting position; (5) the participant puts their body weight on the reaching leg during maximum reach; (6) and significant swinging of the upper limbs during the test. If the test was abandoned, a new round of testing was conducted until the participant completed three successful trials in each direction [30].

### 2.3. Fatigue Protocol

To induce fatigue, participants were asked to perform squatting until exhaustion at a controlled pace of 2 s per squat. Participants were instructed to perform the following steps: Start with fully extended knees, standing upright, arms akimbo. Then, bend the knees to approximately 130° for the squatting motion and then extend the knee to the starting position for under 2 s. A metronome was used to give feedback to the participants and ensure the pace of squatting [31,32]. During the implementation of the fatigue protocol, participants’ heart rates and Rating of Perceived Exertion on the Borg Scale (6–20 scale) were recorded. The fatigue protocol was stopped when all of the following criteria were met: 1. The participant’s heart rate exceeded 90% of their age-calculated maximum heart rate (HRmax = 220). 2. The participant was unable to maintain the original squat frequency. 3. The Borg Scale rating exceeded RPE > 17 (very hard) [33]. In our study, each participant performed a minimum of 50 squats [34]. To ensure that participants were still in a fatigued state, the YBT was immediately repeated after finishing the squats. The fatigue protocol used focused on muscle fatigue in the lower limbs, and the motions of the squat in the fatigue protocol were similar to those of supporting the leg on the dominant side during YBT.

### 2.4. Instruments

During the testing process, a total of 38 optical markers with a diameter of 14 mm were used. These markers were attached to the anatomical locations of the participants based on the Gait2392 muscle-skeletal model, and the same operator consistently placed the markers. The movement trajectories of the markers were recorded using an eight-camera Vicon motion capture system (Vicon Metrics Ltd., Oxford, UK) at a sampling frequency of 1000 frames per second [35,36]. Ground reaction forces and COP measurements were obtained using an embedded Kistler force platform (Kistler Force Platform System 92-81B, Winterthur, Switzerland) fixed in the center of the floor, with a sampling frequency of 200 Hz [37]. Before each experiment, the cameras and laboratory setup were calibrated to ensure stable marker trajectories and minimize noise interference. All participants were instructed to wear standardized tight shorts and were barefoot during the testing process.

### 2.5. Data Processing and Analysis

The standardization of YBT scores was calculated by dividing each reach distance by the participant’s leg length and multiplying it by 100 [22]. 

To optimize the model for individual segment lengths, a static standing pose was recorded before recording marker displacements during YBT. The marker trajectories were smoothed with a 12 Hz low-pass Butterworth filter [38]. The angular displacement of the joints was calculated for each stride using inverse kinematics in Opensim (SimTK v. 4.0.1). The standard inverse dynamics method was utilized to calculate the joint movements, and the joint work was computed based on both inverse kinematics and inverse dynamics. All joint dynamics data were normalized by the participant’s body weight [39]. Subsequently, the kinematic and dynamics parameters of the joints were normalized into 101 data points. COP path, distance, and the area of the circle, which contains 95% of the data, were calculated from the force plate data using a custom-written Matlab script [38]. The kinematic and dynamics data sets were time-normalized to the stance phase (0–100%) for each participant, allowing for time-series comparisons. The dynamics variables were normalized to body weight, and the COP was adjusted for leg length to minimize the influence of height [40]. 

### 2.6. Statistical Analysis

The experimental data were processed using SPSS 26.0 software (IBM, Armonk, NY, USA). The Shapiro–Wilk test was applied to ensure the normality of the data. Paired-sample *t*-tests were used to compare the kinematic, dynamics, and COP activities of the supporting leg before and after fatigue. A significance level of *p* ≤ 0.05 was considered statistically significant. The data are presented as mean values ± standard deviation [25].

## 3. Results

### 3.1. Score of the YBT

Before and after fatigue, the scores of the YBT in three directions are shown in Table 1.

The research findings indicated that after completing a squat fatigue protocol there was a decrease in YBT scores among adult amateur athletes. Specifically, after fatigue, there was a significant decrease in YBT scores in all three directions, i.e., the anterior direction (*p* < 0.001), the posterolateral direction (*p* < 0.001), and the posteromedial direction (*p* = 0.001).

### 3.2. Hip, Knee, and Ankle Joint Angles

Before and after the fatigue protocol, the kinematic results of the hip, knee, and ankle joints in the YBT in three directions are shown in Table 2.

The study results indicated the following: In the anterior direction, there was a significant decrease in hip joint range of motion (*p* < 0.001) and knee joint range of motion (*p* < 0.001) after fatigue compared to before fatigue. In the posterolateral direction, there was a significant decrease in hip joint range of motion (*p* < 0.001) and knee joint range of motion (*p* < 0.001) after fatigue compared to before fatigue. In the posteromedial direction, there was a significant decrease in the hip joint range of motion (*p* = 0.001) and knee joint range of motion (*p* = 0.014) after fatigue compared to before fatigue.

### 3.3. Hip, Knee, and Ankle Joint Torques

**Figure 2:**
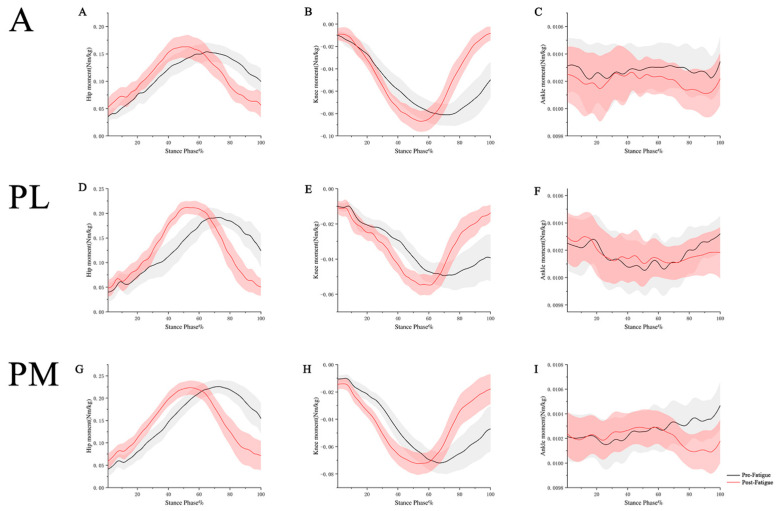
Joint torque results for hip, knee, and ankle during YBT. A: anterior. PL: posterolateral. PM: posteromedial. (**A**): changes in hip torques before and after fatigue in the A direction. (**B**): changes in knee torques before and after fatigue in the A direction. (**C**): changes in ankle torques before and after fatigue in the A direction. (**D**): changes in hip torques before and after fatigue in the PL direction. (**E**): changes in knee torques before and after fatigue in the PL direction. (**F**): changes in ankle torques before and after fatigue in the PL direction. (**G**): changes in hip torques before and after fatigue in the PM direction. (**H**): changes in knee torques before and after fatigue in the PM direction. (**I**): changes in ankle torques before and after fatigue in the PM direction.

Before and after the fatigue protocol, the joint torque results of the hip, knee, and ankle joints in the YBT in three directions are shown in Figure 2 and Table 3.

The study results revealed the following: In the anterior direction, there was a significant increase in peak hip joint torque after fatigue compared to before fatigue (*p* = 0.049). In the posterolateral direction, there was a significant increase in peak hip joint torque after fatigue compared to before fatigue (*p* = 0.001). In the posteromedial direction, there was a significant decrease in peak hip joint torque (*p* = 0.004) and peak ankle joint torque (*p* = 0.001) after fatigue compared to before fatigue.

### 3.4. Hip, Knee, and Ankle Joint Work

**Figure 3:**
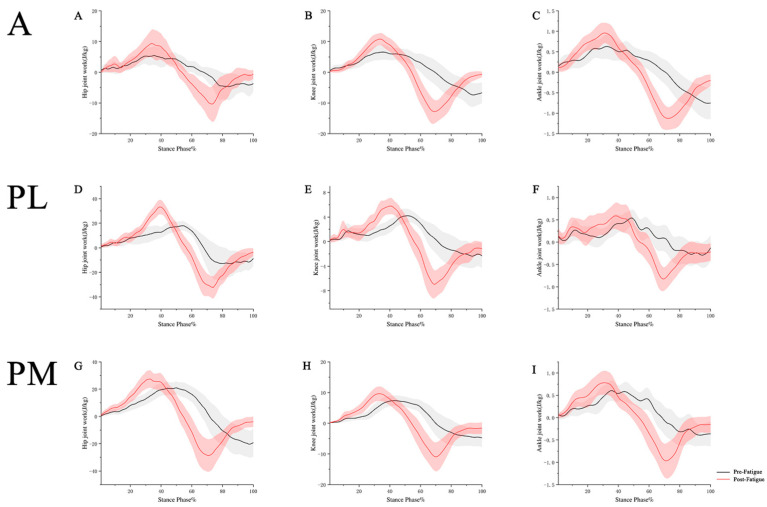
Joint work results for hip, knee, and ankle during YBT. A: anterior. PL: posterolateral. PM: posteromedial. (**A**): changes in hip joint work before and after fatigue in the A direction. (**B**): changes in knee joint work before and after fatigue in the A direction. (**C**): changes in ankle joint work before and after fatigue in the A direction. (**D**): changes in hip joint work before and after fatigue in the PL direction. (**E**): changes in knee joint work before and after fatigue in the PL direction. (**F**): changes in ankle joint work before and after fatigue in the PL direction. (**G**): changes in hip joint work before and after fatigue in the PM direction. (**H**): changes in knee joint work before and after fatigue in the PM direction. (**I**): changes in ankle joint work before and after fatigue in the PM direction.

Before and after the fatigue protocol, the positive work and negative work results of the hip, knee, and ankle joints in the YBT in three directions are shown in Figure 3 and Table 4.

In terms of positive joint work: In the anterior direction, the hip joint showed a significant increase in positive joint work after fatigue compared to before fatigue (*p* < 0.001). The knee joint also exhibited a significant increase in positive joint work after fatigue compared to before fatigue (*p* = 0.009). Similarly, the ankle joint showed a significant increase in positive joint work after fatigue compared to before fatigue (*p* < 0.001). In the posterolateral direction, the hip joint displayed a significant increase in positive joint work after fatigue compared to before fatigue (*p* < 0.001). The knee joint also demonstrated a significant increase in positive joint work after fatigue compared to before fatigue (*p* < 0.001). Additionally, the ankle joint showed a significant increase in positive joint work after fatigue compared to before fatigue (*p* < 0.001). In the posteromedial direction, the hip joint exhibited a significant increase in positive joint work after fatigue compared to before fatigue (*p* = 0.044). The knee joint showed a significant increase in positive joint work after fatigue compared to before fatigue (*p* = 0.007). Similarly, the ankle joint displayed a significant increase in positive joint work after fatigue compared to before fatigue (*p* = 0.007).

In terms of negative joint work: In the anterior direction, the hip joint showed a significant increase in negative joint work after fatigue compared to before fatigue (*p* = 0.001). Similarly, the ankle joint exhibited a significant increase in negative joint work after fatigue compared to before fatigue (*p* = 0.001). In the posterolateral direction, the knee joint displayed a significant increase in negative joint work after fatigue compared to before fatigue (*p* < 0.001). Additionally, the ankle joint demonstrated a significant increase in negative joint work after fatigue compared to before fatigue (*p* < 0.001). In the posteromedial direction, the knee joint exhibited a significant increase in negative joint work after fatigue compared to before fatigue (*p* = 0.011). Similarly, the ankle joint showed a significant increase in negative joint work after fatigue compared to before fatigue (*p* = 0.001).

### 3.5. The COP of YBT

**Figure 4:**
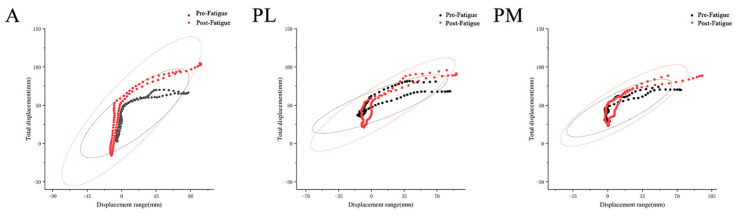
COP motion trajectory during YBT. A: anterior. PL: posterolateral. PM: posteromedial.

Before and after the fatigue protocol, the movement trajectories of the COP in the YBT in three directions, as well as the range of motion in the *x*-axis displacement, are shown in Figure 4 and Table 5.

The research findings indicated that fatigue leads to an increase in the maximum displacement range of the COP in the lower extremities. Before and after fatigue, there were statistical differences in the maximum displacement range of the lower extremities in the anterior direction (*p* = 0.006). However, there were no statistical differences in the maximum displacement range in the posterolateral direction (*p* = 0.064). In the posteromedial direction, there was a statistical difference in the maximum displacement range of the lower extremities (*p* = 0.023).

## 4. Discussion

This study aimed to investigate the performance and changes in lower extremity biomechanical parameters, including kinematics and dynamics, during a YBT in three directions (A, PL, and PM), before and after fatigue, among adult recreational athletes. Specifically, the study focused on the effects of fatigue on hip, knee, and ankle joint ROM, joint torque, joint work, and COP of the dominant supporting leg in the three YBT directions (A, PL, and PM).

In terms of the impact of fatigue on athletes’ YBT performance, this study found that fatigue resulted in a decrease in YBT scores in all three directions among adult recreational athletes, which is consistent with the findings of William Johnston et al. [22]. The decline in YBT performance before and after the test demonstrates that fatigue has a negative effect on the dynamic balance ability of adult recreational athletes.

This study observed that after fatigue, the ROM in the hip and knee joints of the dominant supporting leg significantly decreased during YBT testing in all three directions, and this is consistent with our hypothesis. Previous research by Gribble et al. [41,42] found that individuals with chronic ankle instability had reduced hip and knee flexion ROM during the SEBT, resulting in decreased reach distances and compromised dynamic postural control. Aminaka et al. [43] also discovered that individuals with patellofemoral pain syndrome had lower hip and knee flexion ROM during the SEBT, leading to inferior reach distances compared to a control group. These studies suggest that hip and knee joint ROM can influence SEBT performance and dynamic stability of the lower extremity. Since the YBT is a simplified version of the SEBT, changes in hip and knee joint ROM can similarly affect YBT performance and lower extremity stability. Previous research has shown that fatigue can alter the activity of proximal muscles in the lower extremity, limiting hip and knee joint ROM and subsequently reducing SEBT performance [44]. This is consistent with the findings of our study, suggesting that the decreased hip and knee joint ROM in the sagittal plane of the dominant supporting leg after fatigue is a contributing factor to the observed decrease in YBT performance and dynamic balance ability in the participants.

Previous research has shown that trunk movement in the sagittal plane has an impact on the joint torque of the lower extremities during squatting exercises [45]. Leaning the trunk forward shifts the ground reaction force vector forward, increasing the load on the hip extensors and resulting in a higher hip joint torque. Conversely, maintaining an upright trunk has the opposite effect, reducing the load on the hip extensors and decreasing the hip joint torque [46]. In our experiment, we observed that after fatigue, participants exhibited increased forward trunk inclination during testing in the anterior- and posterior-lateral directions, while decreased forward trunk inclination was observed during testing in the posterior-medial direction. This may explain why, after fatigue, there was an increase in hip joint torque of the dominant supporting leg in the anterior- and posterior-lateral directions, while a decrease in hip joint torque was observed in the posterior-medial direction. Additionally, a decrease in ankle joint torque was observed in the posterior-medial direction after fatigue. Previous studies have indicated that the activation level of the calf muscles, specifically the peroneal muscles, significantly decreases during YBT testing after fatigue [25]. Therefore, we speculate that this decrease in ankle joint torque could be attributed to a reduced activity level of the calf muscles, particularly the peroneal muscles, resulting in insufficient force production to maintain normal joint torque control at the ankle joint.

In terms of joint work, our study revealed that the hip, knee, and ankle joints exhibited negative work throughout the testing process of the YBT, indicating energy absorption. Previous research has shown that fatigue alters the distribution of energy absorption among the lower extremity joints [47]. Our study’s findings support the principle of energy conservation, as the joint work in the lower extremity during the YBT process adhered to this principle [48]. Post-fatigue conditions showed a decrease in proximal hip joint work and a significant increase in knee and ankle joint work compared to pre-fatigue conditions, which aligns with previous research. Both positive and negative work performed by the three lower extremity joints increased overall compared to pre-fatigue conditions. Majid Fatahi et al. [25] suggested that fatigue leads to a decrease in muscle activity associated with joint movement during YBT. We speculate that after fatigue, the lower limb muscles may not generate sufficient force to maintain the body’s original balance and control, requiring increased muscular work to sustain stability in the lower extremity joints and counteract the effects of fatigue on dynamic balance.

The research findings demonstrate that fatigue has a significant impact on increasing the postural sway of lower limbs, with an approximately 15% increase in lower limb sway after fatigue. This finding is consistent with the results of a study by Massimiliano Pau et al. [49]. Previous studies by Penedo et al. [50] have also suggested that fatigue leads to decreased lower limb muscle strength and proprioceptive deficits, impairing the ability of the lower limbs to maintain pre-fatigue stability. This results in an increased range of COP displacement, further compromising postural control. From a biomechanical standpoint, maintaining a larger symmetric stance can keep the center of mass and COP away from the limits of stability, thus aiding in balance control. However, during the YBT test, body posture is inherently positioned in a suboptimal symmetric position, relying solely on one leg to maintain balance. Fatigue exacerbates the asymmetry of body posture [50,51], leading to increased COP oscillations and further reducing the dynamic balance ability of the lower limbs.

During the YBT process, we observed significant effects of fatigue on lower extremity biomechanical parameters. Considering the impact of fatigue on muscles, we recommend athletes enhance training focused on the lower limb muscles during balance training. Previous research has demonstrated that after a period of neuromuscular training, athletes showed remarkable improvements in dynamic postural control [52]. The athletes can adopt a combined approach of resistance training and strength training [53], supplemented with muscle stretching exercises [54], specifically targeting the lower limb muscles to enhance lower limb stability and reduce the risk of sports injuries.

However, our study also has some limitations. Firstly, the study mainly focused on adult male recreational athletes, and there may be physiological and biomechanical differences between genders and age groups. Future research should include female athletes and individuals from different age groups to gain a more comprehensive understanding of the effects of the YBT on lower extremity biomechanics in diverse populations. Secondly, our study only concentrated on the dominant leg, neglecting the study of the non-dominant leg. Since athletes typically use both legs during training and competition, understanding the performance of the non-dominant leg in balance training is also essential. Finally, our research only explored lower extremity biomechanical parameters during the YBT, while other factors such as neural control and the impact of vestibular sensations on balance ability are worth further exploration. Therefore, future research should consider a broader range of factors to comprehensively understand the effects of balance training and YBT testing on athletes.

## 5. Conclusions

Our research findings indicate that fatigue significantly impacts the dynamic balance of athletes’ lower limbs. Post-fatigue, there was a decrease in hip and knee joint angles in all three directions. There was an increase in hip joint movements in the anterior- and posterior-lateral directions, while a decrease in hip and ankle joint movements was observed in the posterior-medial direction. Additionally, there was an increasing trend in positive and negative joint work of the hip, knee, and ankle joints after fatigue. The range of COP displacement also increased. Based on the research findings, we recommended that athletes avoid engaging in activities requiring frequent rapid changes of direction, acceleration, and deceleration after experiencing fatigue. Such activities could include lateral cutting movements in soccer or sudden stops and turns in basketball. This caution was due to the fact that fatigue reduces lower limb balance and stability. Performing these high-intensity movements while fatigued might further increase the risk of sports-related injuries. Furthermore, in their daily balance training and rehabilitation routines, athletes should closely monitor their fatigue levels. They should also engage in exercises aimed at enhancing the stability of the lower limb joints and muscle strength. Examples of such exercises included Pilates or specific balance-oriented routines. By adopting targeted measures, athletes can effectively mitigate the adverse impact of fatigue on their body balance, enhance their athletic performance, and concurrently reduce the risk of sports injuries.

## Figures and Tables

**Table 1:** Score of the YBT.

	Pre-Fatigue (Mean ± SD)	Post-Fatigue (Mean ± SD)
A (%)	37.96 ± 4.86	32.36 ± 4.43 *
PL (%)	69.65 ± 8.61	64.93 ± 8.77 *
PM (%)	66.80 ± 6.24	63.87 ± 5.98 *

Note: * indicates significant difference from before fatigue.

**Table 2:** Sagittal range of motion of hip, knee, and ankle during YBT.

		Pre-Fatigue	Post-Fatigue
A (°)	Hip	24.13 ± 4.88	15.96 ± 3.45 *
	Knee	68.42 ± 5.04	58.47 ± 3.36 *
	Ankle	30.55 ± 9.36	31.49 ± 7.01
PL (°)	Hip	69.60 ± 6.52	61.59 ± 5.84 *
	Knee	59.28 ± 4.68	50.80 ± 4.83 *
	Ankle	20.96 ± 6.76	21.71 ± 4.36
PM (°)	Hip	66.26 ± 8.13	60.32 ± 9.99 *
	Knee	69.27 ± 8.33	65.86 ± 7.52 *
	Ankle	26.64 ± 7.89	26.75 ± 6.17

Note: * indicates significant difference from before fatigue.

**Table 3:** Peak joint torques of hip, knee, and ankle during YBT.

		Pre-Fatigue	Post-Fatigue
A (Nm/kg)	Hip	0.16 ± 0.03	0.17 ± 0.04 *
	Knee	−0.09 ± 0.02	−0.09 ± 0.02
	Ankle	0.01 ± 0.0003	0.01 ± 0.0002
PL (Nm/kg)	Hip	0.22 ± 0.02	0.23 ± 0.02 *
	Knee	−0.06 ± 0.01	−0.06 ± 0.01
	Ankle	0.01 ± 0.0003	0.01 ± 0.0003
PM (Nm/kg)	Hip	0.25 ± 0.02	0.24 ± 0.03 *
	Knee	−0.08 ± 0.01	−0.08 ± 0.01
	Ankle	0.0108 ± 0.0003	0.0106 ± 0.0003 *

Note: * indicates significant difference from before fatigue.

**Table 4:** Mean positive work and negative work of hip, knee, and ankle during YBT.

		Pre-Fatigue	Post-Fatigue
A (J/kg)	Hip PW	4.05 ± 2.14	5.40 ± 2.90 *
	Hip NW	−5.05 ± 3.49	−5.75 ± 3.63 *
	Knee PW	4.88 ± 1.66	5.74 ± 1.49 *
	Knee NW	−6.02 ± 3.57	−6.96 ± 2.71
	Ankle PW	0.50 ± 0.15	0.59 ± 0.15 *
	Ankle NW	−0.55 ± 0.30	−0.70 ± 0.19 *
PL (J/kg)	Hip PW	11.74 ± 5.19	15.13 ± 2.76 *
	Hip NW	−15.64 ± 12.42	−18.71 ± 4.68
	Knee PW	2.56 ± 0.55	3.14 ± 0.62 *
	Knee NW	−2.64 ± 1.89	−4.27 ± 1.38 *
	Ankle PW	0.41 ± 0.085	0.48 ± 0.124 *
	Ankle NW	−0.41 ± 0.17	−0.55 ± 0.12 *
PM (J/kg)	Hip PW	13.41 ± 3.74	14.96 ± 4.74 *
	Hip NW	−16.21 ± 4.70	−17.63 ± 4.89
	Knee PW	4.42 ± 1.09	5.01 ± 1.25 *
	Knee NW	−5.20 ± 3.77	−6.88 ± 2.85 *
	Ankle PW	0.44 ± 0.084	0.49 ± 0.14 *
	Ankle NW	−0.43 ± 0.26	−0.63 ± 0.21 *

Note: PW: positive work; NW: negative work; * indicates significant difference from before fatigue.

**Table 5:** Maximum displacement range of lower limbs during YBT.

	Pre-Fatigue	Post-Fatigue
A (mm)	125.98 ± 64.36	147.53 ± 65.87 *
PL (mm)	131.36 ± 64.13	143.47 ± 67.49
PM (mm)	111.03 ± 58.27	128.32 ± 59.91 *

Note: * indicates significant difference from before fatigue.

## Data Availability

The data are unavailable due to privacy or ethical restrictions.
